# Relative Mutant Allele Frequency Role in ctDNA-Guided Anti–Epidermal Growth Factor Receptor Rechallenge in Metastatic Colorectal Cancer

**DOI:** 10.1200/PO-26-00199

**Published:** 2026-09-01

**Authors:** Jesús Yaringaño, Patricia García Pastor, Francesc Salva, Nadia Saoudi González, Javier Ros, Iosune Baraibar, Marta Rodríguez Castells, Clara Salva de Torres, Pau Mascaró Baselga, Ariadna García, Caterina Vaghi, Ana Vivancos, Josep Tabernero, Elena Elez

**Affiliations:** ^1^ Medical Oncology Department, Vall d’Hebron University Hospital, Barcelona, Spain; ^2^ Colorectal Cancer Group, Digestive Tumors Group, Vall d’Hebron Institute of Oncology, IIS IR-HUVH, CIBERONC, Barcelona, Spain; ^3^ Cancer Genomics Lab, Vall d'Hebron Institute of Oncology, IIS IR-HUVH, Barcelona, Spain

## Introduction

Treatment options for metastatic colorectal cancer (mCRC) after failure of standard fluoropyrimidine-, oxaliplatin-, and irinotecan-based therapies remain limited, with objective response rates (ORR) generally below 10% and median survival typically shorter than 1 year despite recent approvals of trifluridine-tipiracil plus bevacizumab combination and fruquintinib.^1-3^ Within this landscape of limited late-line efficacy, anti–epidermal growth factor receptor (anti-EGFR) rechallenge has emerged as a biologically rational and clinically meaningful strategy for selected patients.^4,5^

The concept arises from the observation that resistant *RAS*-, *BRAF*-, or *EGFR*-mutant tumor clones, emerging under selective anti-EGFR pressure, tend to decay over time once the drug is withdrawn.^6,7^ Serial circulating tumor DNA (ctDNA) analyses have demonstrated that these resistance mutations exhibit a median half-life of 4-5 months after discontinuation, potentially restoring tumor sensitivity to EGFR blockade.^4,7^

Phase II CHRONOS and CRICKET trials demonstrated that, in patients with ctDNA-confirmed *RAS/BRAF* wild-type (wt) status, anti-EGFR rechallenge alone or in combination with irinotecan can achieve response rates up to 30%, median progression-free survival (mPFS) around 4 months, and median overall survival (mOS) exceeding 12 months.^8,9^ A recent pooled analysis including four prospective Italian studies further supported the clinical activity of anti-EGFR rechallenge in ctDNA-selected *RAS*/*BRAF* wt mCRC.^10^

Increasing assay sensitivity has progressively revealed ultra–low-frequency resistant subclones whose biological and clinical significance remain uncertain. Early BEAMing-based analyses and subsequent retrospective studies from the CRYSTAL and CO.17 trials suggested that some low-allele-frequency resistant alterations may not fully preclude sensitivity to EGFR blockade, raising the possibility that clonal burden, rather than mutation detection alone, may influence therapeutic resistance.^11-13^ Contemporary sequencing platforms increasingly detect extremely low-frequency variants whose biological significance may differ substantially according to clonal dominance and genomic context.^14,15^ Therefore, binary interpretation of resistance alterations as simply mutant or wild-type may inadequately reflect the functional relevance of subclonal populations.

In this report, we present two real-world cases illustrating that liquid biopsy–guided anti-EGFR rechallenge may remain a biologically rational strategy despite low-mutant allele frequency (MAF) resistance mutations. To better interpret these findings, we introduce the concept of relative MAF (rMAF), defined as the ratio of the resistance mutation MAF and the highest MAF detected in the same sample.^16^ All identifying information has been removed. According to institutional ethics committee policy and local regulations, informed consent is not required for retrospective anonymized case reports involving deceased patients.

### Case 1

A 60-year-old man with no relevant medical history was diagnosed with left-sided *RAS/BRAF* wt, microsatellite stable mCRC with liver metastases (M1). First-line treatment with fluorouracil, leucovorin, and oxaliplatin (FOLFOX) plus panitumumab achieved partial response (PR) after 12 cycles. Following multidisciplinary evaluation, the patient underwent resection of the primary tumor and liver M1, with no evidence of residual disease after surgery.

Approximately 5 months after surgery, liver and lung progression (PD) was documented. Reintroduction of fluoropyrimidine plus panitumumab achieved renewed disease control; however, treatment interruptions related to skin toxicity and infectious complications were associated with subsequent pulmonary PD. FOLFOX plus panitumumab was restarted, later followed by 5-fluorouracil and panitumumab maintenance.

After prolonged benefit from anti–EGFR-based therapy (63 cycles), the patient experienced pulmonary, bone, and hepatic PD, leading to the switch to second-line fluorouracil, leucovorin, and irinotecan (FOLFIRI) plus aflibercept. After cycle 3, he was admitted for significant toxicity with consequent treatment interruption due to deterioration in performance status. At subsequent reassessment, hepatic and pulmonary progression was documented.

**FIG 1.**
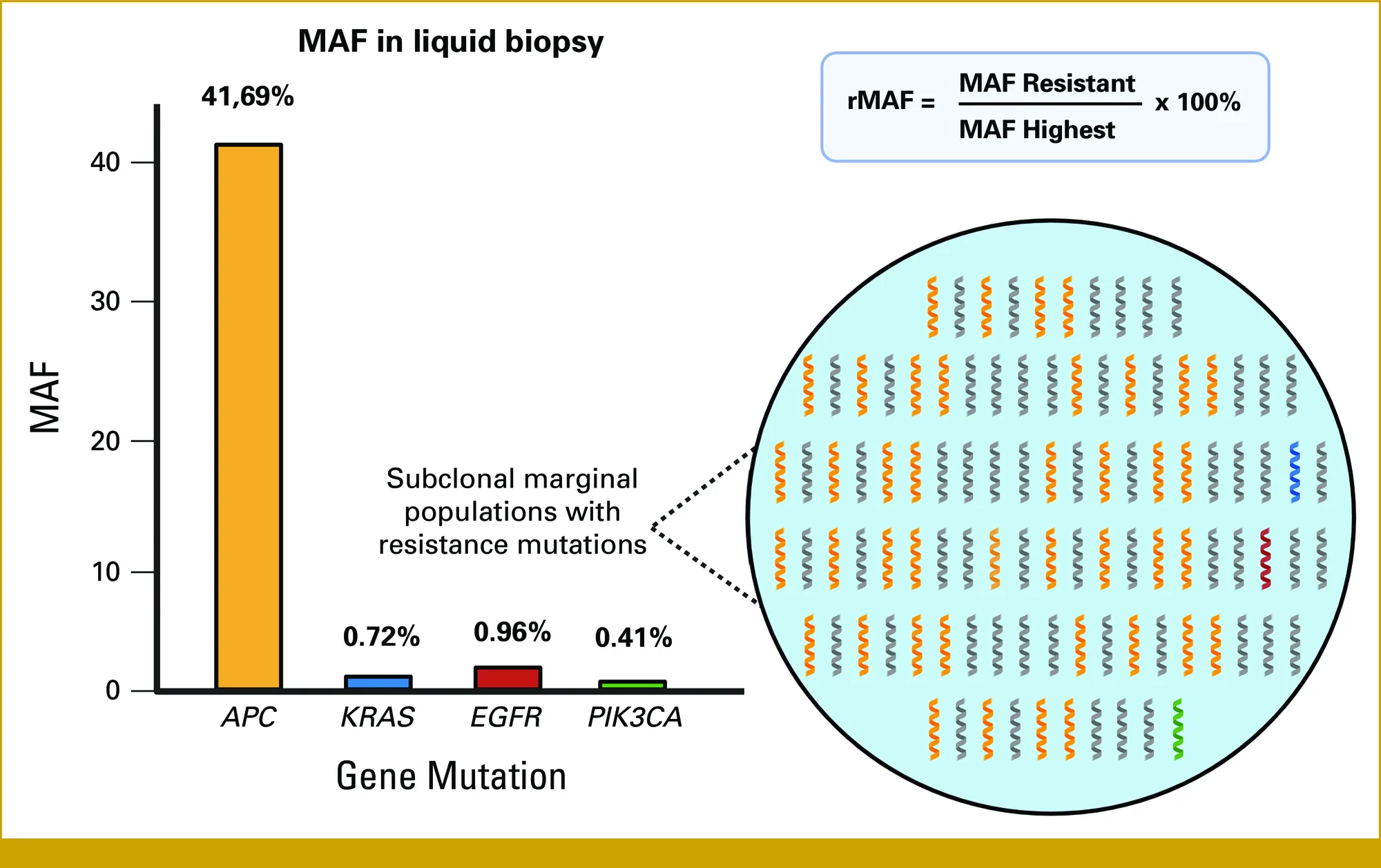
Definition of rMAF in circulating tumor DNA. Relative mutant allele frequency is defined as the ratio between the MAF of a resistance-associated alteration and the highest MAF detected in the same ctDNA sample, representing a surrogate measure of the clonal weight of resistance mutations relative to truncal tumor alterations. Values shown correspond to case 1. MAF, mutant allele frequency; rMAF, relative mutant allele frequency.

At this moment, ctDNA profiling using the Guardant 360 CDx assay detected low-frequency resistance mutations in *EGFR* (MAF 0.96%), *KRAS* (MAF 0.72%), and *PIK3CA* (MAF 0.41%), whereas *APC* represented the dominant alteration (MAF 41.69%), corresponding to rMAF <1% (Fig 1).

**FIG 2.**
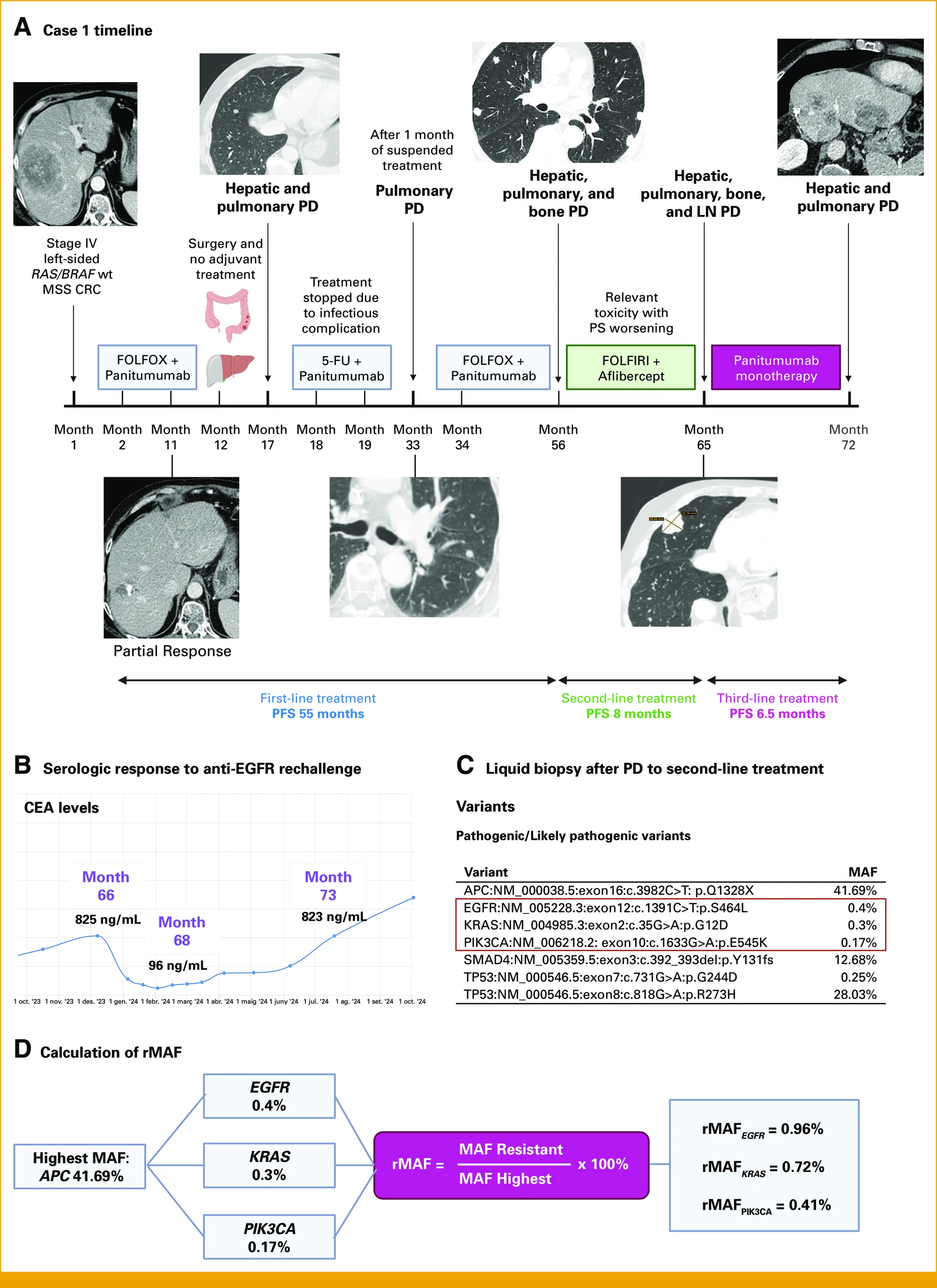
Clinical course and molecular profiling of case 1. (A) Timeline summarizing systemic treatments received, radiologic assessments by CT progression events, and progression-free survival for each treatment line. (B) Longitudinal serum CEA levels during anti-EGFR rechallenge until disease progression. (C) Circulating tumor DNA profiling using the VHIO360 (Guardant) panel showing detected resistance-associated mutations at the time of rechallenge. (D) Calculation of rMAF for the three resistance-associated mutations identified in ctDNA. CEA, carcinoembryonic antigen; CT, computed tomography; EGFR, epidermal growth factor receptor; rMAF, relative mutant allele frequency.

Given the prolonged prior sensitivity to anti-EGFR therapy, the 9-month anti–EGFR-free interval, limited alternative therapeutic options, and the low clonal weight of resistance-associated alterations, anti-EGFR rechallenge with panitumumab monotherapy was initiated. After six cycles, radiologic reassessment demonstrated PR accompanied by a marked decrease in carcinoembryonic antigen levels (Fig 2). Following cycle 12, disease progression was observed in liver, lymph nodes, and lungs. Best supportive care treatment was decided.

### Case 2

A 55-year-old man with no significant medical history was diagnosed with left-sided *RAS*/*BRAF* wt mCRC presenting with bowel obstruction, intestinal perforation, and liver metastases. The patient underwent emergency surgery (pT3N1b).

First-line treatment was administered within an academic clinical trial. The patient was randomly assigned to receive FOLFOX plus panitumumab, followed by FOLFIRI plus bevacizumab upon disease progression. Although following cycle 6 he achieved PR, disease remained unresectable. Progression involving liver and lymph nodes subsequently occurred after prolonged disease control (PFS, 17 months). Second-line FOLFIRI plus bevacizumab also achieved PR before later hepatic progression (PFS, 7 months).

During screening for a subsequent clinical trial, ctDNA analysis (Guardant 360 CDx, Guardant Health, Inc, Redwood City, CA) detected low-frequency alterations in *PIK3CA* E545K (MAF 0.01%) and *BRAF* V600E (0.002%), whereas *APC* and *TP53* were detected at 0.07%. A contemporaneous metastatic liver biopsy analyzed using VHIO300, an in-house 435-gene capture panel (Illumina, Barcelona, Spain), revealed mutations in *APC* (MAF 78%), *PIK3CA* E545K (MAF 5%), and *TP53* (MAF 73%). The tumor was HER2-negative, and polymerase chain reaction testing for *KRAS* and *BRAF* V600E was negative.

The patient subsequently received treatment with anti–PD-1, anti–cytotoxic T-cell lymphocyte-4, and MEK inhibition within a clinical trial for approximately 13 months, when he developed liver and nodal PD.

**FIG 3.**
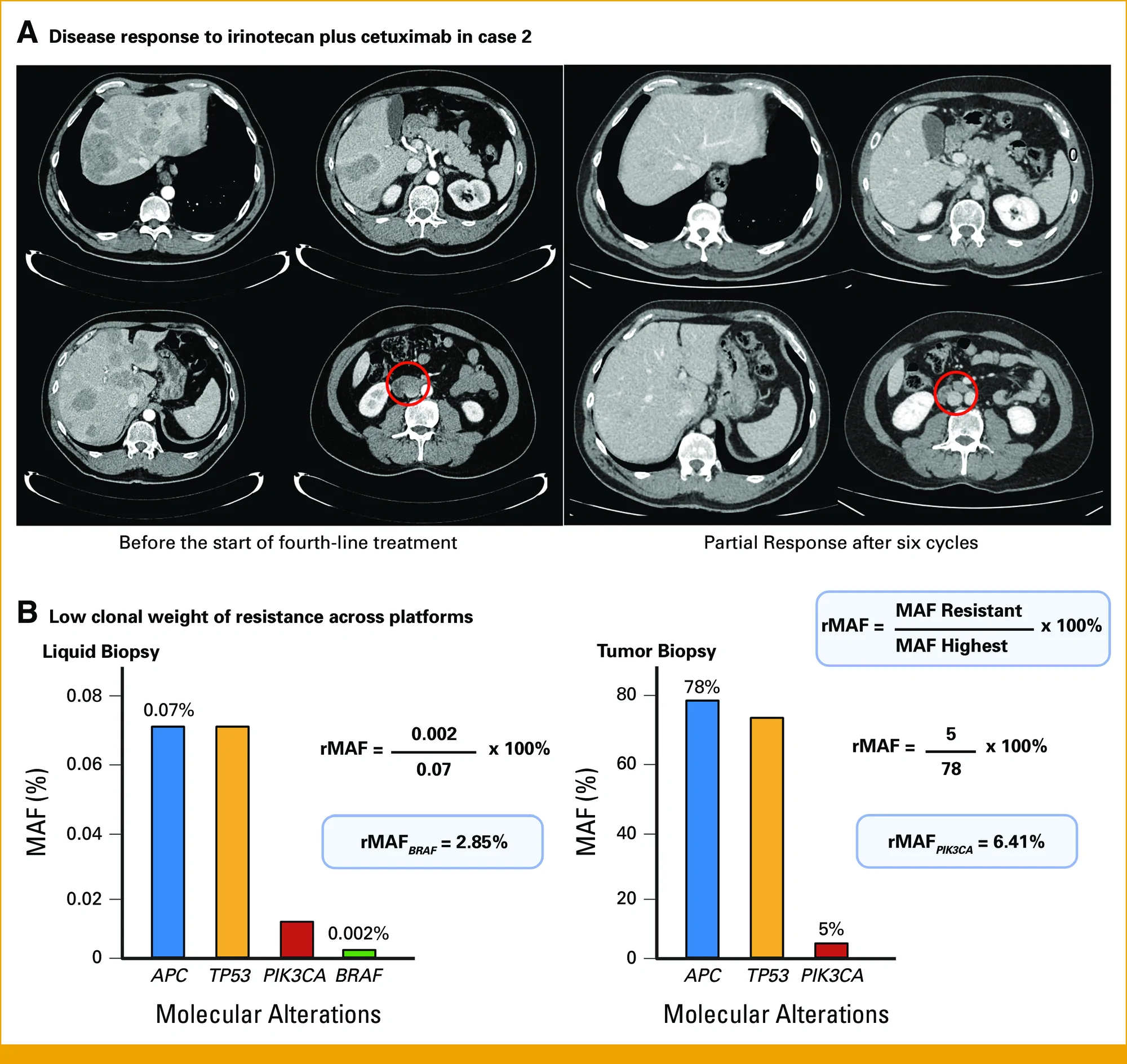
Radiologic response and molecular characterization of case 2. (A) Computed tomography images showing baseline tumor burden before initiation of irinotecan plus cetuximab (left) and partial radiologic response after six treatment cycles (right). (B) Resistance-associated mutations identified by circulating tumor DNA and tissue-based sequencing, including calculation of rMAFs for *BRAF* in liquid biopsy and *PIK3CA* in tissue biopsy. rMAF, relative mutant allele frequency.

At the time of progression after immunotherapy-based treatment, in the context of the patient's marked historical sensitivity to anti-EGFR therapy, prolonged anti–EGFR-free interval, and the extremely low clonal representation of *BRAF* V600E in plasma (rMAF 2.85% relative to APC), cetuximab rechallenge was administered in combination with irinotecan, consistent with previously reported rechallenge strategies. Radiologic reassessment after six cycles demonstrated PR (Fig 3). Disease progression occurred after approximately 11 months.

The patient subsequently received three additional lines of therapy without clinical benefit and later died from progressive disease.

## Discussion

The advent of ctDNA profiling has enabled real-time monitoring of clonal kinetics, transforming anti-EGFR rechallenge from an empirical clinical decision into a biomarker-informed strategy that accounts for the heterogeneous and dynamic nature of tumor evolution.^17^

Notably, recent randomized clinical data underscore the critical role of ctDNA in patient selection. The FIRE-4 trial did not show a statistically significant survival benefit with cetuximab rechallenge when applied empirically in baseline *RAS* wt tumors, highlighting that a substantial proportion of patients harbor resistance mutations after an anti–EGFR-free window therapy.^18^ In contrast, the ctDNA-guided CITRIC trial demonstrated that excluding patients with detectable resistance alterations and restricting rechallenge to those with *RAS*/*BRAF* and *EGFR* extracellular domain (ECD) wt ctDNA enriches for a population with improved disease control and PFS, supporting the concept that refined molecular hyperselection may enhance the likelihood of benefit.^19^

Highly sensitive ctDNA assays increasingly identify ultra–low-frequency resistant subclones whose capacity to drive phenotypic resistance remains uncertain.^11^ In this context, the two cases presented expand the traditional framework of ctDNA interpretation. We hypothesize that the quantitative context of detected mutations, particularly their rMAF, may hold relevant biological and clinical information. Low-frequency resistance variants detected at MAF levels substantially below truncal mutations (eg, *APC* or *TP53*) may represent minor subclones with limited phenotypic dominance.^16^

In case 1, ctDNA revealed low-frequency *EGFR*, *KRAS*, and *PIK3CA* mutations, with much higher *APC* MAF (41.7%). The resulting rMAF <1% suggested biologically marginal subclonal populations. Guided by this interpretation and a 9-month anti–EGFR-free interval, panitumumab monotherapy was initiated. In case 2, an ultra-low *BRAF* V600E signal (MAF 0.002%; rMAF 2.85% relative to *APC*) on liquid biopsy, together with prior EGFR sensitivity, supported the decision to initiate cetuximab plus irinotecan rechallenge. In both patients, anti–EGFR-based rechallenge was associated with radiologic responses and clinically meaningful disease control despite multiple prior therapies, leading to the hypothesis that relative, rather than absolute, MAF interpretation may help contextualize low-level resistance signals during therapeutic decision making.^16,20^

Our observations are consistent with recent real-world evidence. In a retrospective cohort of 60 patients with *RAS*/*BRAF* wt mCRC undergoing anti-EGFR rechallenge, approximately 30% harbored ctDNA-detected resistance mutations in *RAS*, *BRAF*, or *EGFR* ECD, generally present at very low allele frequencies (median MAF 0.36%, median rMAF 1.69%). Importantly, the mere presence of low-frequency resistance mutations did not completely abrogate clinical activity, as ORR and disease control rates (DCRs) were comparable between mutation-negative and mutation-positive patients (ORR 21% *v* 17%; DCR 61% *v* 56%). However, significant differences emerged after stratification by rMAF. Patients with rMAF ≤12.4% appeared to derive greater benefit, with longer mPFS (6.1 *v* 2.6 months; *P* = .04) and numerically prolonged mOS (28.3 *v* 3.7 months, *P* = .09) compared with patients harboring higher rMAF values.^16^

These data reinforce the notion that not all detectable resistance variants exert equivalent biological pressure. Clonal representation of resistance-associated alterations may better capture the functional significance of subclones and their true impact on treatment sensitivity.^9,10^

This report is limited by its retrospective nature. Moreover, serial plasma sampling immediately before rechallenge was not available in case 2. Nevertheless, these cases illustrate the potential complexity of interpreting low-frequency resistance-associated alterations detected by highly sensitive ctDNA assays and support further evaluation of rMAF in prospective rechallenge studies.

Incorporating rMAF into ctDNA interpretation may help contextualize the biologic relevance of low-frequency resistance-associated alterations, refining patient selection for anti-EGFR rechallenge.
